# Treating Depression with Transcutaneous Auricular Vagus Nerve Stimulation: State of the Art and Future Perspectives

**DOI:** 10.3389/fpsyt.2018.00020

**Published:** 2018-02-05

**Authors:** Jian Kong, Jiliang Fang, Joel Park, Shaoyuan Li, Peijing Rong

**Affiliations:** ^1^Psychiatry Department, Massachusetts General Hospital, Harvard Medical School, Charlestown, MA, United States; ^2^Guang’anmen Hospital, China Academy of Chinese Medical Sciences, Beijing, China; ^3^Institute of Acupuncture and Moxibustion, China Academy of Chinese Medical Sciences, Beijing, China

**Keywords:** vagus nerve, transcutaneous vagus nerve stimulation, transcutaneous auricular vagus nerve stimulation, depression, brain network, anti-inflammation

## Abstract

Depression is a highly prevalent disorder, and its treatment is far from satisfactory. There is an urgent need to develop a new treatment for depression. Although still at its early stage, transcutaneous auricular vagus nerve stimulation (taVNS) has shown promising potential for treating depression. In this article, we first summarize the results of clinical studies on the treatment effect of taVNS on depression. Then, we re-analyze a previous study to identify the specific symptoms taVNS can relieve as indicated by subscores of the 24-item Hamilton Depression Scale in patients with depression. We found that taVNS can significantly reduce multiple symptoms of depression patients, including anxiety, psychomotor retardation, sleep disturbance, and hopelessness. Next, we pose several hypotheses on the mechanism of taVNS treatment of depression, including directly and indirectly modulating the activity and connectivity of key brain regions involved in depression and mood regulation; inhibiting neuro-inflammatory sensitization; modulating hippocampal neurogenesis; and regulating the microbiome–brain–gut axis. Finally, we outline current challenges and lay out the future directions of taVNS treatment of depression, which include (1) intensively comparing stimulation parameters and “dose effect” (treatment frequency and duration) to maximize the treatment effect of taVNS; (2) exploring the effect of taVNS on disorders comorbid with depression (such as chronic pain disorders, cardiovascular disorder, and autism) to provide new “two-for-one” treatment approaches for patients with these disorders; and (3) applying multiple scale methods to explore the underlying mechanism of taVNS.

## Introduction

The vagus nerve (VN) is the longest cranial nerve in the human body and is involved in the regulation of multiple systems (1). Due to this wide influence on multiple systems and its important role in maintaining homeostasis, stimulating the VN to modulate the function of related organs has long drawn the attention of investigators (2). As a slow-acting therapy, cervical vagus nerve stimulation (VNS) has been approved by the US Food and Drug Administration for managing treatment-refractory epilepsy in 1997 and for chronic treatment-resistant depression in 2005 (1). However, surgical risks, technical challenges, and potential side effects have limited the application of VNS (3, 4).

To overcome such barriers of applying invasive VNS (iVNS), non-invasive transcutaneous vagus nerve stimulation (tVNS) methods have been developed. Currently, there are two main ways to apply tVNS. One is to superficially apply stimulation on the cervical nerve using a specially designed device, such as GammaCore, and the other is to apply stimulation on the ear. In this paper, we will focus on the latter. The rationale of tVNS on the ear (transcutaneous auricular VNS, taVNS) is based on anatomical studies demonstrating that certain parts of the ear area (concha and lower half of the back ear over the mastoid process) have afferent VN distribution (5–7). According to the “bottom-up” mechanism of the central nervous system (CNS), electrical stimulation of these areas may produce activity changes in the VN pathway in the brain stem and central structures (8), producing a modulation effect similar to iVNS (9–11). taVNS has been used to treat disorders, such as epilepsy (12, 13), pre-diabetes (14), depression, and chronic tinnitus (15), as well as to boost associative memory (16).

In this manuscript, we summarize the findings of clinical studies on taVNS treatment of depression, re-analyze a previous data set to explore the specific symptoms tVNS can relieve in patients with depression [as indicated by Hamilton Depression Rating Scale (HAMD) subscores] (17), and discuss the potential underlying mechanism, limitations, and future direction of taVNS. Please also see recent review articles on iVNS treatment of depression (2), iVNS/taVNS treatment of chronic pain (18), clinical application (19), and efficacy and tolerability (20).

## Potentials of taVNS Treatment of Depression and its Side Effects

Major depressive disorder (MDD) is a highly prevalent disorder that can significantly reduce quality of life (21). Current treatments for MDD are far from satisfactory (22–24), thereby calling for new treatments for MDD. As a non-invasive peripheral neuromodulation method, taVNS may be a promising treatment option for patients with MDD.

The first taVNS clinical trial on individuals with MDD was performed by Hein and colleagues (9). They investigated the treatment effect of bilateral taVNS on MDD patients using an add-on design (antidepressant therapy with real or sham taVNS). They found that compared to the sham group, the real taVNS group showed significant improvement on the Beck Depression Inventory after a 2-week treatment (five times per week). However, there was no significant difference on the HAMD between the two groups.

In a subsequent non-randomized clinical study with 160 MDD patients (17), we investigated the taVNS treatment effect by training the patients to apply bilateral taVNS at home. The first cohort of patients (*n* = 91) received taVNS for 12 weeks; the second cohort (*n* = 69) first received 4 weeks of sham taVNS followed by 8 weeks of real taVNS. After the fourth week, patients in the taVNS group had greater decreases in the 24-item HAMD score and higher rates of good responders than those of the sham taVNS group. The clinical improvements continued until week 12.

In a recent single-arm study (25), Trevizol and colleagues recruited 12 patients with MDD and tested the effect of taVNS on the bilateral mastoid process (10-session taVNS over 2 weeks). The results showed that 17-item HAMD scores were reduced significantly after the 2-week treatment. All patients exhibited a clinical response, defined as a reduction of HAMD scores of at least 50%. The effect remained 1 month after treatment.

Although the above studies suggest that taVNS can reduce the symptoms of MDD, no study has reported how it can modulate the specific symptoms of MDD patients. To address the question, we re-analyzed the data of our previous study (17) and explored how taVNS can modulate HAMD subscores of patients with MDD (Table 1) by performing a repeated measurement analysis with Bonferroni correction to adjust the *p*-value (0.05/7 = 0.007 significance level). We found that compared with sham taVNS, 1-month taVNS can significantly reduce multiple symptoms of MDD patients, including anxiety, psychomotor retardation, sleep disturbance, and hopelessness. We also observed a downward trend in cognitive disturbance and diurnal variation (Table 1).

**Table 1:** Pre- and post-treatment differences in HAMD subscores between real and sham transcutaneous auricular vagus nerve stimulation (taVNS) cohorts; *p* values indicating significant difference after Bonferroni correction (*p* = 0.05/7 = 0.007) are marked in bold.

HAMD item	Group	*N*	Pre-treatment (Mean ± SD)	Post-treatment (Mean ± SD)	Post–Pre (Mean ± SD)	Effect size	*p*-Value
Anxiety	taVNS	88	7.2 ± 2.6	5.4 ± 2.4	−1.7 ± 2.4	0.565	**0.001**
	staVNS	60	6.6 ± 1.9	6.0 ± 2.1	−0.6 ± 1.7		

Weight	taVNS	88	0.3 ± 0.6	0.1 ± 0.4	−0.1 ± 0.7	0.025	0.888
	staVNS	60	0.4 ± 0.6	0.3 ± 0.5	−0.1 ± 0.5		

Cognitive disturbance	taVNS	88	4.0 ± 2.7	2.3 ± 1.8	−1.8 ± 2.3	0.458	0.010
	staVNS	60	3.6 ± 1.9	2.7 ± 1.4	−0.9 ± 1.4		

Diurnal variation	taVNS	88	1.2 ± 1.1	0.7 ± 0.9	−0.5 ± 1.2	0.412	0.017
	staVNS	60	0.9 ± 1.0	0.9 ± 1.0	−0.0 ± 1.0		

Psychomotor retardation	taVNS	88	4.9 ± 1.7	3.1 ± 1.7	−1.8 ± 1.8	0.717	**<0.001**
	staVNS	60	4.6 ± 1.3	3.9 ± 1.4	−0.7 ± 1.1		

Sleep disturbance	taVNS	88	4.0 ± 1.9	2.3 ± 1.7	−1.7 ± 1.7	0.575	**0.001**
	staVNS	60	4.1 ± 1.9	3.4 ± 1.9	−0.8 ± 1.5		

Hopelessness	taVNS	88	3.6 ± 1.6	2.0 ± 1.3	−1.5 ± 1.8	0.635	**<0.001**
	staVNS	60	4.1 ± 1.4	3.5 ± 1.6	−0.6 ± 1.2		

Transcutaneous auricular vagus nerve stimulation is a quite safe and well-tolerable treatment method (20). Reported mild/moderate side effects include tinnitus or acceleration of original tinnitus and local problems at stimulation sites, such as pain, paresthesia, or pruritus during or after stimulation (17, 26). Since there are no direct fibers connecting the ear VN to the heart (27, 28), both left and right ears should be safe for applying taVNS. In a recent study (28), Kreuzer et al. measured EKG changes after 24 months of taVNS and found that taVNS has no arrhythmic effects on cardiac function in tinnitus patients with no known pre-existing cardiac pathology. In another study on taVNS treatment of MDD (9), investigators also found that heart rate, blood pressure, and blood test values did not change over the 2-week treatment period.

Interestingly, applying taVNS on the bilateral mastoid process (25) seems to be associated with more severe side effects as compared to taVNS applied on the concha (9, 17). In the Trevizol study (25), of the total 12 patients, 10 patients reported mild to moderate diurnal sleepiness after stimulation, six reported mild to moderate tension headaches with no need for medication, and four reported mild to moderate nausea. We speculate this may be due to the electrical current flowing across the whole brain during bilateral stimulation. Further study is needed to explore the side effects of taVNS on the bilateral mastoid process.

## Mechanisms/Hypothesis on taVNS Treatment of Depression

### taVNS Can Modulate the Brain Network Associated with the Neuropathology of Depression

**Figure 1:**
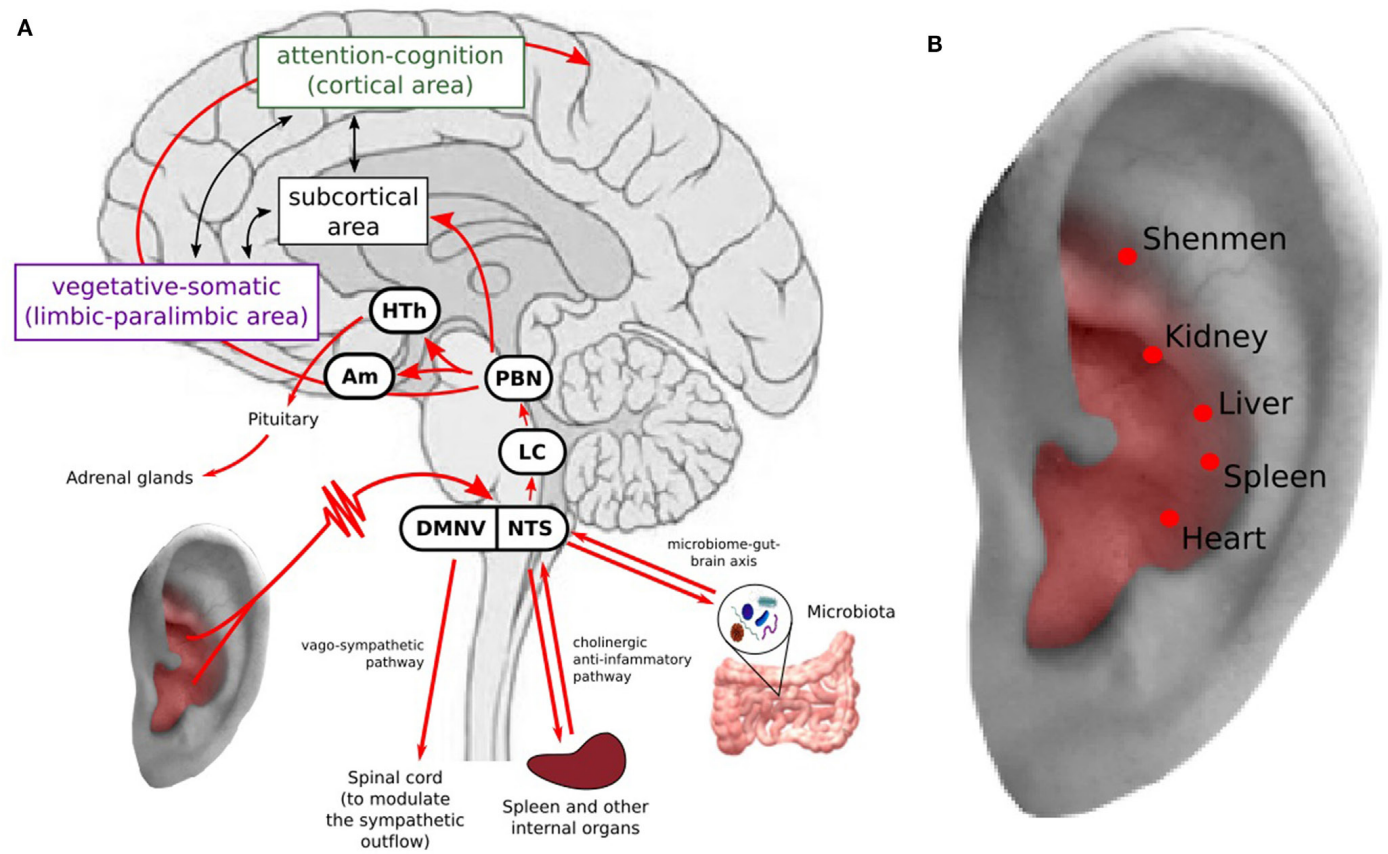
**(A)** Transcutaneous auricular vagus nerve stimulation (taVNS) can modulate the brain network associated with the neuropathology of depression and inhibit inflammation response. Stimulation of the auricular branch of the vagus nerve (VN, indicated in red), which projects to the nucleus tractus solitari (NTS), continuing to the locus coeruleus and parabrachial nucleus. From the parabrachial nucleus, it propagates to various brain regions involved in depression (39, 40). taVNS may inhibit inflammation response to relieve stress and depressive symptoms. HTh, hypothalamus; PBN, parabrachial nucleus; LC, locus coeruleus; NTS, nucleus tractus solitary; DMNV, dorsal motor nucleus of the vagus nerve. **(B)** Auricular acupuncture points used for treating depression and other mental disorders at area with VN distribution.

A growing body of evidence has shown that depression is associated with structural and functional abnormalities in multiple brain regions involved in emotional processing, self-representation, reward, and external stimulus (stress, distress) interactions (29–37). Based on the limbic-cortical dysregulation hypothesis (38–40), the brain regions involved in MDD are associated with two components: the vegetative-somatic component, including the subgenual cingulate cortex, anterior insula, hippocampus, hypothalamus, and amygdala, and the attention-cognition component, including the dorsal frontal area, dorsal cingulate cortex, inferior parietal cortex, and posterior cingulate cortex. Located between the two components are the basal ganglia and thalamus, which closely communicate with the two components (Figure 1A).

Neural anatomy has shown that the auricular branch of the vagus nerve (ABVN) projects to the nucleus tractus solitari (NTS), which is further connected with other brain regions, such as the locus coeruleus, parabrachial nucleus, hypothalamus, thalamus, amygdala, hippocampus, anterior cingulate cortex (ACC), anterior insula, and lateral prefrontal cortex (19, 41). Thus, the VN has direct and indirect connections to the depression-related cortical–limbic-thalamic–striatal neural circuits, influencing the activity of these regions (42–46) (Figure 1A).

Recent neuroimaging studies (47–53) found that compared with a control condition, taVNS stimulation can produce activation of the “classical” central vagal projections, e.g., widespread activity in the NTS, dorsal raphe, locus coeruleus, parabrachial area, hypothalamus, amygdala, ACC, anterior insula, and nucleus accumbens. For instance, in a recent study (53), we found that taVNS produced fMRI signal increases in the anterior insula compared to sham stimulation in patients with MDD. The insula activation level during the first stimulation session in the taVNS group was significantly associated with clinical improvement after 4 weeks, as shown by the reduction of HAMD scores. In addition, we found that after 1 month of taVNS treatment, resting-state functional connectivity (rsFC) between the default mode network (DMN), a key network involved in depression (54–60), and the anterior insula and parahippocampus decreased, while the FC between the DMN and the orbital prefrontal cortex and precuneus increased compared with sham taVNS (61). In another study using the same dataset, we found that taVNS can significantly increase rsFC between the right amygdala and left dorsolateral prefrontal cortex compared with sham taVNS (62). These results further endorse the extensive modulation effect of taVNS on brain regions involved in depression.

### taVNS May Relieve Symptoms of Depression by Modulating the Inflammation System

Literature suggests that stress initiates cognitive, affective, and possibly biological processes that increase risk for depression (63, 64). Inflammation may play an important role in this process. Specifically, neuro-inflammatory sensitization provoked by stress elicits profound changes in behavior, including common symptoms of depression such as sad mood, anhedonia, fatigue, psychomotor retardation, and social–behavioral withdrawal (63–66). In this process, the hypothalamus, anterior insula, and ACC play an important role (63).

Studies have suggested that the VN plays a crucial role in bidirectionally connecting the brain and immune system, reducing exacerbated inflammation processes outside the CNS (67). Specifically, the VN may participate in the modulation of the inflammation system through two pathways: (1) activating the hypothalamic–pituitary–adrenal axis and suppressing peripheral inflammation *via* glucocorticoids (68) and (2) through the mechanism of the “inflammatory reflex” (67, 69–72) (Figure 1A). In the inflammatory reflex, accumulation of inflammatory cytokines activates VN fibers from which afferent signals ascend to the NTS (69). The NTS projects to efferent vagal neurons in the dorsal motor nucleus of the VN, which projects to intrinsic ganglia in the viscera such as in the spleen and liver. Then, acetylcholine is released in the parenchyma of target organs, activating local nAChRa7 macrophages. Production of inflammatory cytokines is inhibited, attenuating the activity of the immune system (73). In addition, VNS may also trigger the vago-sympathetic pathway, i.e., vagal afferents in the NTS trigger the dorsal motor nucleus of the vagus nerve to modulate the sympathetic outflow by innervating preganglionic sympathetic neurons in the spinal cord (74, 75) (Figure 1A).

### Other Potential Mechanisms

Recently, accumulating evidence has demonstrated that microbe interactions are crucial in maintaining homeostasis in humans. Studies (76–79) have suggested that gut microbiota can influence brain function, mood, and behavior by interacting with the central nervous system through neural, endocrine, and immune pathways. Particularly, studies have shown that the microbiota is crucial in modulating the stress response and stress-related behaviors, such as depression and anxiety (76, 78–80). It is well-known that the VN can significantly modulate the gastrointestinal, immune, and endocrine systems (1). Thus, taVNS may also regulate the functions of the above systems and achieve a treatment effect in depression by adjusting the microbiome–brain–gut axis (80) (Figure 1A).

Also, based on the neurogenic theory of depression (81), depression results from impaired adult hippocampal neurogenesis, and restoration of adult hippocampal neurogenesis leads to recovery. Studies have shown that VNS may stimulate hippocampal neurogenesis, providing another possible mechanism for depression treatment. For instance, studies have shown that VNS can alter the transmission of neurotransmitters, such as serotonin and norepinephrine, which can modulate hippocampal cell proliferation (2). Thus, taVNS may also relieve depression symptoms by modulating hippocampal neurogenesis (2).

### taVNS and Auricular Acupuncture—Old Wine in a New Bottle

Stimulating certain areas on the ear to treat disorders is not something new. Acupuncture, an ancient therapeutic method, has a long history of applying stimulation on different parts of the body, including the ear, to treat disorders. Nowadays, auricular acupuncture has become a crucial school of acupuncture and is widely used in acupuncture practice (82). Nevertheless, the underlying mechanism of auricular acupuncture remains unclear.

Transcutaneous auricular vagus nerve stimulation provides a new angle to understand auricular acupuncture (83). For instance, the auricular acupoints used for depression are also located at the area with VN distribution (Figure 1B), Thus, auricular acupuncture and taVNS perform the same or similar treatment procedure guided by different theories. Usichenko et al. found that the analgesic effects of auricular acupuncture may be explained by stimulation of ABVN (83). Further study to verify the specificity of auricular acupuncture will not only deepen our understanding of auricular acupuncture, but also facilitate the development of taVNS and peripheral neuromodulation.

## Challenges and Future Directions

### Where to Stimulate and How to Stimulate

A neural anatomy study (6) showed that the auricular branch of the VN is mainly distributed on the concha (including the outer auditory canal) and lower half of the back ear. Thus, these areas should be the target of taVNS. Nevertheless, given that the branching of the nerve in the concha is variable across individuals and there are other nerve branches in the area, it remains a challenge to stimulate VN consistently across different individuals.

In a recent study, Kraus et al. (49) compared taVNS-evoked fMRI signal changes at the anterior and posterior sides of the left outer auditory canal. Many brain regions excluding the insular cortex showed fMRI signal changes. The fMRI signals were notably decreased in the parahippocampal gyrus, posterior cingulate cortex, and right thalamus (pulvinar) following anterior auditory canal wall stimulation (49). In another brain imaging study (84), the authors compared the fMRI signal changes evoked by 25 Hz stimulation at the inner tragus, inferoposterior wall of the ear canal, cymba concha, and earlobe (control location without VN distribution). The results showed that stimulation at the inner tragus and cymba concha produced significantly greater activation in the NTS and LC compared with the control location (earlobe). Further ROI analysis showed that only stimulating the cymba concha produced a significantly stronger activation in both the NTS and LC than stimulating the control location.

These results suggest that taVNS at different locations of the ear with VN innervation may modulate different brain pathways, which may be associated with different modulation effects. More studies are needed to systemically investigate the linkage between the brain regions and different ear areas.

Stimulation frequency and intensity are both crucial parameters in taVNS. One may imagine that low-frequency stimulation (2–10 Hz) is not as efficient as higher frequency stimulation (20–30 Hz) which is currently used in iVNS for epilepsy and depression. In reality, investigators have used different frequencies in previous studies with wide ranges [1.5 Hz (9), 20 Hz (17), and 120 Hz (25)].

Studies suggest that different stimulation frequencies could produce different brain changes and neurotransmitter releases (85, 86). In an animal study (87), investigators found that the anti-epileptic effect of 20 Hz taVNS was significantly longer than those of 2 and 100 Hz as measured by the duration of seizure suppression. A recent study (88) on taVNS treatment of drug-resistant epilepsy showed a significant reduction in seizure frequency in patients of the 25 Hz group as compared to the 1 Hz group. However, in another study (26) on migraine patients, investigators found that 1 Hz taVNS produced greater improvement than 25 Hz taVNS. Taken together, these studies imply that the optimal stimulation frequency may vary depending on the disorder.

Likewise, there are few systematic studies on the optimal intensity of taVNS. Previous studies have suggested that stimulation intensity could be set to a level that could arouse a tingling but tolerable sensation (17, 61, 62). In addition, the intensity may interact with frequency [individuals with low frequency stimulation tend to be able to tolerate higher stimulus intensities than those who receive high frequency stimulation (88)]. However, investigators (9) have applied subthreshold taVNS (the patients could not feel the sensation) and relieved symptoms in patients with MDD, which calls for further research on this topic.

Finally, very few studies have been carried out to explore the “dose effect” of taVNS, i.e., how long and how frequently we should apply taVNS. iVNS stimulation usually lasts for many hours per day. Such durations are unrealistic for taVNS. Current studies range from 30-min stimulation durations two times per day (17) to 15-min stimulation durations five times per week (9). Also, if the patients were trained to apply the taVNS by themselves, the problem of compliance is difficult to evaluate and may somehow counterbalance the interest for such a technique.

In summary, investigators have used a wide range of stimulation parameters in taVNS treatment of depression. Identifying the optimal stimulation parameters and “dose” may represent the crucial next step for taVNS research.

### Future Directions

(1) Although previous studies have suggested that taVNS holds potential for patients with MDD, the key parameters and “dose” that can maximize the treatment effect remain unknown. Studies to directly compare different stimulation parameters (frequency and intensity), duration, and frequency of treatment are needed. In addition, large randomized clinical trials are also needed to test the treatment effect of taVNS on patients with different age ranges (from children and teenagers to older adults), as well as different depression severities, so that we can have a better idea of the target population for taVNS.
(2) Depression can be comorbid with many other disorders, such as chronic pain (89, 90), cardiovascular disorder (91, 92), inflammatory bowel disease (93), irritable bowel syndrome (94), and autism (95). Thus, it may also provide a new treatment option for “two-for-one” treatment approaches for patients with disorders comorbid with depression.
(3) Multiple scale mechanism studies incorporating brain imaging tools, inflammation markers, vagal tone measurements, and neural transmitters are needed to deepen our understanding of taVNS and facilitate development of new treatment methods for depression and disorders comorbid with depression.

In summary, taVNS can significantly reduce anxiety, retardation, sleep disturbance, and hopelessness symptoms in patients with depression. Current literature suggests that it may relieve the symptoms of MDD through multiple mechanisms. Further research is needed to identify the optimal stimulation parameters and “dose” of taVNS, testing its effect on MDD patients of different ages and severities, as well as on disorders with comorbid depression.

## Author Contributions

JK, PR, and JF conceived the idea; JK, PR, JF, JP, and SL did the literature search and prepared figures and manuscript.

## Conflict of Interest Statement

JK holds equity in a startup company (MNT) and pending patents to develop new neuromodulation devices. All other authors declare no competing financial interests.
